# Urinary Levels of *N*-Nitroso Compounds in Relation to Risk of Gastric Cancer: Findings from the Shanghai Cohort Study

**DOI:** 10.1371/journal.pone.0117326

**Published:** 2015-02-06

**Authors:** Ling Xu, Yong-Hua Qu, Xin-Di Chu, Renwei Wang, Heather H. Nelson, Yu-Tang Gao, Jian-Min Yuan

**Affiliations:** 1 Masonic Cancer Center, University of Minnesota, Minneapolis, Minnesota, United States of America; 2 Division of epidemiology and community health, University of Minnesota, Minneapolis, Minnesota, United States of America; 3 Robert R. Stempel College of Public Health and Social Work, Department of Dietetics and Nutrition, Florida International University, Miami, FL, United States of America; 4 Shanghai Cancer Institute and Cancer Institute of Shanghai Jiaotong University, Shanghai, China; 5 Division of Cancer Control and Population Sciences, University of Pittsburgh Cancer Institute, Pittsburgh, Pennsylvania, United States of America; 6 Department of Epidemiology, Graduate School of Public Health, University of Pittsburgh, Pittsburgh, Pennsylvania, United States of America; National Health Research Institutes, TAIWAN

## Abstract

**Background:**

*N*-Nitroso compounds are thought to play a significant role in the development of gastric cancer. Epidemiological data, however, are sparse in examining the associations between biomarkers of exposure to *N*-nitroso compounds and the risk of gastric cancer.

**Methods:**

A nested case-control study within a prospective cohort of 18,244 middle-aged and older men in Shanghai, China, was conducted to examine the association between urinary level of *N*-nitroso compounds and risk of gastric cancer. Information on demographics, usual dietary intake, and use of alcohol and tobacco was collected through in-person interviews at enrollment. Urinary levels of nitrate, nitrite, *N*-nitroso-2-methylthiazolidine-4-carboxylic acid (NMTCA), *N*-nitrosoproline (NPRO), *N*-nitrososarcosine (NSAR), *N*-nitrosothiazolidine-4-carboxylic acid (NTCA), as well as serum *H. pylori* antibodies were quantified in 191 gastric cancer cases and 569 individually matched controls. Logistic regression method was used to assess the association between urinary levels of *N*-nitroso compounds and risk of gastric cancer.

**Results:**

Compared with controls, gastric cancer patients had overall comparable levels of urinary nitrate, nitrite, and *N*-nitroso compounds. Among individuals seronegative for antibodies to *H. pylori*, elevated levels of urinary nitrate were associated with increased risk of gastric cancer. The multivariate-adjusted odds ratios for the second and third tertiles of nitrate were 3.27 (95% confidence interval = 0.76–14.04) and 4.82 (95% confidence interval = 1.05–22.17), respectively, compared with the lowest tertile (*P* for trend = 0.042). There was no statistically significant association between urinary levels of nitrite or *N*-nitroso compounds and risk of gastric cancer. Urinary NMTCA level was significantly associated with consumption of alcohol and preserved meat and fish food items.

**Conclusion:**

The present study demonstrates that exposure to nitrate, a precursor of *N*-nitroso compounds, may increase the risk of gastric cancer among individuals without a history of *H. pylori* infection.

## Introduction

Despite the decrease in incidence and mortality rates of stomach cancer worldwide over the past three decades, gastric cancer is the fourth most commonly diagnosed cancer and the third most common cause of cancer death [1]. Even if the current trend in rate decline continues, this malignancy will remain as one of the most common cancers worldwide due to aging of current populations in high-risk regions [2]. A distinguishing feature of gastric cancer is the remarkable geographic variation in incidence and mortality rates worldwide. Eastern Asia has the highest incidence rate of gastric cancer with more than 60 per 100,000 person-years, significantly higher than the rates in North America and Africa which are below 9 per 100,000 [3]. The considerable decrease in the incidence rates of gastric cancer among Japanese migrants to the United States relative to their counterparts in Japan in the past several decades suggest that environmental factors play a significant role in the development of gastric cancer [4]. Hence, identification of environmental risk factors for gastric cancer would inform strategies for primary prevention against this malignancy.

*N*-Nitroso compounds (NOCs) have shown carcinogenic effects in experimental studies. Approximately 300 NOCs have been tested for carcinogenicity in laboratory experiments, with 90% of them demonstrating carcinogenic effects across different animal species, including higher primates [5, 6]. Certain NOCs have been classified as “probably carcinogenic to humans” by the International Agency for Research on Cancer (IARC) [7]. According to the 2010 IARC report, ingested nitrate or nitrite under conditions that result in endogenous nitrosation is probably carcinogenic to humans [8]. Humans are exposed to NOCs from exogenous sources and of endogenous synthesis. Exogenous NOCs are directly derived from certain types of food, such as processed meat, salted or smoked fish, and pickled and dried vegetables [9]. Available data suggest that NOCs in food are found more frequently, and at higher concentration, in Asia than Western countries [10]. Endogenous NOCs are formed from nitrosation of secondary amines or amides by N_2_O_3_ and (H_2_NO_2_) ^+^, both of which are nitrite-derived nitrosation agents. Nitrite could derive either directly from food, or indirectly from reduction of nitrate by oral and enteric bacteria [11–14]. Endogenous formation of NOCs is probably catalyzed by the heme iron present in red meat [15, 16]. In humans, nitrosation takes place in more acidic environment such as the stomach, especially where antioxidant vitamin C level is low, than in neutral environment [17]. Nitrosation reactions can be enhanced by certain bacteria as well as under certain inflammatory conditions such as oxidative burst [18]. Approximately 45%-75% NOCs that humans are exposed to is derived from endogenous synthesis [19]. Individuals with high exposure to NOCs are hypothesized to be at increased risk of developing gastric cancer.

Epidemiological studies examining the association between NOCs or NOC-containing food and gastric cancer risk have produced inconsistent results [20]. These inconsistencies were likely due to measurement error in the assessment of exposure to NOCs in most, if not all, epidemiological studies as well as to the lack of information on cofactors that have impact on the endogenous nitrosation [8]. A biomarker approach that assesses total NOCs (i.e. the sum of both exogenous and endogenous sources) would overcome some of these limitations and increase the validity of results. In the present study, we quantified urinary levels of nitrate and nitrite, two precursors of NOCs, as well as *N*-nitroso-2-methylthiazolidine-4-carboxylic acid (NMTCA), *N*-nitrosoproline (NPRO), *N*-nitrososarcosine (NSAR), and *N*-nitrosothiazolidine-4-carboxylic acid (NTCA) 4 major non-volatile NOCs present in human urine [19]. The half-life of individual NOCs varies from several hours to several days. The NPRO and NSAR are excreted in urine almost unchanged, and proportional to the orally administered dose in rat model [21]. The *in vivo* administration of nitrate and *N*-nitrosamine precursors has shown high efficiency in the production of NMTCA, NTCA and NPRO [22, 23]. Thus monitoring NPRO or other NOCs excreted in the urine appeared to be a suitable procedure for estimating human daily exposure to both endogenous and exogenous NOCs [21, 22, 24]. This study was nested within the Shanghai Cohort Study, a prospective cohort of 18,244 middle-aged and older men, in Shanghai, China. The primary aim of the present study was to evaluate the association between urinary levels of NOCs and their precursors and risk of gastric cancer. The secondary aim of present study was to investigate the potential modifying effect of infection with *H*. *pylori* bacteria, alcohol consumption, cigarette smoking, and serum/urinary antioxidant measurement on the NOC-gastric cancer association.[25]

## Materials and Methods

### Study Population

The design of the Shanghai Cohort Study has been described in detail elsewhere [26, 27]. Briefly, four small, geographically defined communities over a wide area of the City of Shanghai were selected for a prospective, cancer epidemiologic cohort for study of environmental exposure and cancer. Complete rosters of all residents in these selected communities for identification of eligible subjects were obtained from local police stations. The eligibility criteria were men between 45 and 64 years of age who had no history of cancer. Between January 1986 and September 1989, 18,244 men (∼80% of eligible subjects) participated in the study. Each participant was interviewed in person using a structured questionnaire eliciting information on demographics, use of alcohol and tobacco, usual adult diet, and medical history. At the completion of the interview, a 10-mL nonfasting blood sample and a single-void (i.e., spot) urine sample were collected from each participant. Blood and urine samples were collected usually between 5 pm and 9 pm and placed in an icebox (∼4°C) immediately after collection. Multiple aliquots of serum and urine from each subject were made and stored at -70°C. From each subject one 25 ml vial of urine was mixed with 100 mg sodium hydroxide (NaOH) before it was stored at -70°C for long-term storage. All participants provided their written consent for participation of this study at enrollment. The consent form for interview and collection of biospecimens at baseline was approved by the Institutional Review Board of the Shanghai Cancer Institute, Shanghai China. All surviving cohort participants also provided their written consent to continue to participate in the cohort study during annual follow-up in-person interviews. The consent form for continued participation of this study was approved by the Institutional Review Boards of the Shanghai Cancer Institute and the University of Pittsburgh. This study has been approved by the Institutional Review Boards of the Shanghai Cancer Institute, the University of Minnesota, and the University of Pittsburgh.

Current diet was assessed through a food frequency questionnaire that included 45 food items representing commonly consumed local foods in Shanghai, China, in early 1980’s. Annual follow-up for incident cancers and deaths has been carried out since 1986. All surviving participants were contacted in-person annually for vital status and cancer diagnosis. We also performed record linkage analysis with databases of the Shanghai Cancer Registry and Shanghai Municipal Vital Statistics. By the end of 2012, only 609 (3.3%) original cohort participants were lost to our annual follow-up interview. In addition, 573 (3.1%) subjects refused our request for annual follow-up interviews, although their cancer and vital status have been ascertained and updated through the annual record linkage analyses. Thus the follow-up for incidence of cancer and death among cohort participants was almost complete. The present study included 197 patients with incident gastric cancer whose initial diagnosis was made between date of enrollment and March 1998. Diagnoses of 179 (91%) cancers were based on histopathologic evidence. The remaining 18 (9%) cases were diagnosed based on radiographic imagines with consistent clinical evidence (n = 14) or death certificate only (n = 4). There were 45 patients with cancer at gastric cardia and 146 at non-cardia.

For each case, we chose three control subjects randomly among all eligible participants of the cohort study who met the matching criteria. All three chosen controls were individually matched to index cases by age (±2 years), month and year of biospecimen collection, and neighborhood of residence at recruitment.

### Laboratory Assays

The aliquots of urine samples of the subjects were pulled from the biospecimen repository and then sorted into the matched case-control sets. All 4 urine samples within a given matched case-control set (i.e., 1 case and 3 controls) were arranged in a random order and tested in the same batch for all laboratory measurements. The case/control status of the test urine samples was blind to laboratory personnel.

NSAR, NPRO, NTCA and NMTCA were analyzed by a gas chromatography coupled with thermal energy analyzer (GC-TEA) according to the method described previously [22]. Briefly a 7.5 mL aliquot of NaOH-treated urine was extracted 3 times with 20 mL of methanol-dichloromethane (1:9, v/v) after addition of 75 ng *N*-nitrosopipecolic acid (NPIC) as internal standard, 2.0 g sodium chloride (NaCl), and 1.5 mL 20% ammonium sulfamate solution in 1.8 *M* H_2_SO4. The combined extracts were dried over anhydrous sodium sulfate and concentrated to dryness by rotary evaporator at 30°C, and derivatized in 2 ml ether with excess diazomethane (prepared with 2 g *N*-Methyl-*N*-Nitroso-p-toluenesulfonamide, 60 ml ether, 12 ml of 60% potassium hydroxide and 12 ml of methanol).The methyl ester of the five *N*-nitrosamino acids in ethereal solution were concentrated to 0.1 ml and quantified with a 10 μl aliquot by GC-TEA. For gas chromatography, a glass column (2 m x 3 mm i.d.) packed with 5% FFAP on Chromosorb WHP (80–100 mesh) was used at a temperature of 180°C. The temperature of the injection port of the gas chromatography was 200°C, and the flow rate of the nitrogen carrying gas was 50 ml/min. For the thermal energy analysis, the temperature of the pyrolyzer was 500°C, interface was 200°C, and vacuum to 0.9 mm Hg. The recoveries of NSAR, NPRO, NMTCA, and NTCA added at 30 ug/L each were 75%, 79%, 91% and 96%, respectively. The detection limits ranged from 0.1 to 0.5 μg/L, depending on the compound.

Nitrate and nitrite were analyzed according to the method described previously with some modification [25]. Cadmium was prepared by the reaction of zinc with 20% cadmium sulphate. The ammonium chloride buffer solution was adjusted to pH 9.6–9.7. Urine samples were deproteined before measurement of nitrite and nitrate was performed. Urine sample was adjusted to pH 8–9, the mixture was incubated at 50–60°C for 10 min, then 2 ml 12% ZnSO_4_ solution was added and incubated at 50–60°C for another 10 min, and additional 1 ml 0.5 *N* NaOH was added before the contents cooled to room temperature. After adding 17.6 ml of water, the sample was passed through filter paper and the filtrate was collected after discarding the first 10 ml filtrate. For measurement of nitrite, 5 ml 0.5% sulfanilamide and 2 ml 0.5% *N*-(1-naphthyl) ethylenediamine dihydrochloride was added to the 10 ml aliquot of filtrate described above before the absorbance of the final solution was read at 540 nm. For determination of nitrate, 5 ml NH_4_OH buffer solution and 18 ml water was added to 2 ml filtrate before it passed through a Cd column at 3–5 ml/min. The column was washed with 15 ml water and the combined effluent collected. After adding 5 ml 0.5% sulfanilamide and 2 ml 0.5% N-(1-naphthyl) ethylenediamine dihydrochloride, the absorbance of the final solution was read at 540 nm after standing for 20 min. The final concentrations of nitrite and nitrate were calculated using the standard curve. For quality control purpose, two batches of urine samples with three duplicates each that were blind to laboratory personnel were dispersed among the test samples. The intra- and inter-assay coefficients of variation for all NOCs and their precursors measured were 4–15% and 12–33%%, respectively.

A history of infection with *H*. *pylori* was determined by detection of serum immunoglobulin G (IgG) antibodies to *H*. *pylori* using an enzyme-linked immunosorbent assay (ELISA) described previously [28]. This ELISA was developed and validated using *H*. *pylori* strains that were prevalent in the study population [28]. The methods for quantification of antioxidants including serum carotenoids (i.e., α-carotene, β-carotene, β-cryptoxanthin, lycopene, lutein/zeaxanthin, retinol, α-tocopherol, γ-tocopherol, and vitamin C) and urinary catechins including epigallocatchin (EGC) were described in previous reports of this study population [29, 30].

### Statistical Analysis

Of 197 cases and 591 controls, urine samples of 6 cases (and 18 their matched controls) and additional 4 controls had missing values of one or more urinary measurements. Therefore, the present study included 191 cases and 569 matched control subjects after excluding these subjects with missing values in the primary analyses. In addition, we added urinary nitrite assay late on the study samples and had values of nitrite on 104 cases and 308 matched controls only, on which all statistical analysis involving urinary nitrite was based.

The concentrations of urinary nitrate, nitrite and 4 NOCs measured were expressed as μg/g creatinine to take into account varying water content in urine across different individuals. The distributions of the concentrations of these urinary analytes were markedly skewed with few subjects possessing high values, which were normalized, to a large extent, by their logarithmic transformation. Therefore, formal statistical tests on continuous values of NOCs, nitrate and nitrite were performed on logarithmically transformed values. Spearman rank correlation analysis was performed to assess the relationships between individual urinary NOCs, serum antioxidants and urinary EGC. The χ^2^ test was used to compare the distributions of selected demographics, cigarette smoking, alcohol consumption and IgG antibodies to *H*. *pylori* between cases and controls. The analysis of covariance method was used to examine differences in the geometric mean of urinary nitrate, nitrite and NOCs across different levels of smoking, alcohol intake, or serologic status of *H*. *pylori* infection, intake of fresh dark green vegetables, intake of salted/pickled vegetables, or intake of preserved meats, fish or eggs among control subjects only. The *Wilcoxon* statistic method was used to assess the difference in the distributions of urinary NOCs or their precursors between cases and controls.

Conditional logistic regression method was used to assess the associations between levels of urinary NOCs, nitrate and nitrite and gastric cancer risk [31]. The associations were measured by odds ratios (ORs) and their corresponding 95% confidence interval (CIs) and *P’s* for trends. Study subjects were grouped into three levels (low, intermediate, and high) based on the tertile or high/low distributions of detectable urinary NOCs among all control subjects. The linear trend test for the exposure-disease risk associations was based on ordinal values (0–2) for the three exposure levels. We also examined the NOC-gastric cancer risk association in subgroups stratified by cigarette smoking (ever or never), alcohol consumption (ever or never), serologic status of *H*. *pylori* (negative or positive), levels of serum antioxidants (below and equal to or above median), or level of urinary EGC (below and equal to or above median), as well as by subsite of gastric cancer (cardia and non-cardia). For the subgroup analyses, matched sets were broken and unconditional logistic regression was used to maximize the sample size available for stratified statistical analysis. The matching factors (age, year of biospecimen collection, and neighborhood of residence at recruitment) were included in all unconditional logistic regression models as covariates. The presence of *H*. *pylori* antibodies in serum, cigarette smoking, heavy alcohol consumption, serum levels of β-carotene and vitamin C, and urinary level of EGC were identified risk/protective factors for gastric cancer in this study population [28–30, 32]. These factors were included in the multivariable logistic regression models when examining the independent effect of NOCs on gastric cancer risk.

Statistical computing was conducted using SAS version 9.2 (SAS Institute Inc., Cary, NC). All *P’*s reported are two-sided, and those that were less than 0.05 were considered to be statistically significant.

## Results

The mean age (standard deviation) of case patients at cancer diagnosis was 63.4 (5.6) years. The average time interval between biospecimen collection and cancer diagnosis was 5.1 (3.0) years (ranging from 1 month to 12 years). There was no significant difference in body mass index (kg/m^2^) or level of education between cases and controls. Individuals who developed gastric cancer consumed more cigarettes and alcohol, and were more likely to be seropositive for IgG antibodies to *H*. *pylori* than their matched controls (Table 1).

**Table 1:** Demographic and lifestyle characteristics of gastric cancer patients (cases) and the control subjects (controls), The Shanghai Cohort Study.

	Cases	Controls	2-sided *P* *
Number of subjects	191	569	
Age at interview (year), mean (SD)	58.3 (5.3)	58.3 (5.1)	0.983
Body mass index (kg/m2), mean (SD)	22.5 (3.5)	22.2 (3.1)	0.357
Level of education (%)			
No formal schooling or primary school	71 (37.1)	188 (33.0)	0.288
Middle school	64 (33.5)	175 (30.8)	
High school	22 (11.5)	95 (16.7)	
College graduates or above	34 (17.8)	111(19.5)	
Alcohol drinking on a weekly basis (%)			
Nondrinkers	97 (50.8)	325 (57.1)	0.128
Ever Drinkers	94 (49.2)	244 (42.9)	
No. of alcoholic drinks per day (%)			
Nondrinkers	97 (50.8)	325 (57.1)	0.004
<4	66 (34.5)	205 (36.0)	
4+	28 (14.7)	39 (6.9)	
No. of years of alcohol drinking (%)			
Nondrinkers	97 (50.8)	325 (57.1)	0.157
<20	22 (11.5)	68 (12.0)	
20 to <40	54 (28.3)	116 (20.4)	
40+	18 (9.4)	60 (10.5)	
Cigarette smoking (%)			
Never smokers	59 (30.9)	238 (41.8)	0.027
Former smokers	22 (11.5)	53 (9.3)	
Current smokers	110 (57.6)	278 (48.9)	
No. of years of smoking (%)			
Never smokers	59 (30.1)	238 (41.8)	0.011
<30	52 (27.2)	109 (19.2)	
30+	80 (41.9)	222 (39.0)	
No. of cigarettes per day (%)			
Never smokers	59 (30.9)	238 (41.8)	0.014
<20	69 (36.1)	154 (27.1)	
20+	63 (33.0)	177 (31.1)	
*H*. *pylori* antibody serologic status† (%)			
Negative	20 (11.0)	94 (17.4)	0.041
Positive	162 (89.0)	447 (82.6)	

* Derived from t-test for means or chi-square test for percentage differences between cases and controls.

† Nine cases and twenty eight controls with missing *H*. *pylori* status were excluded from the analysis.

The correlation coefficients among nitrate, nitrite and four NOCs ranged from 0.03 to 0.70 (Table 2). The highest correlation coefficient was for NPRO with NTCA (r = 0.70), followed by NPRO with nitrate (r = 0.50) and by NTCA with NMTCA (r = 0.48). There was no or weak correlation between urinary NOCs or their precursors and antioxidant measurements including urinary EGC and serum carotenoids, retinol, tocopherols and vitamin C (all correlation coefficients < 0.25) (data not shown).

**Table 2:** Spearman correlation coefficients among urinary *N*-nitroso compounds measured among control subjects, The Shanghai Cohort Study.

	NPRO	NSAR	NTCA	Nitrate	Nitrite†
NMTCA	0.29*	-0.07	0.48*	0.20*	-0.07
NPRO	…	0.07	0.70*	0.51*	0.20*
NSAR	…	…	-0.03	-0.08	0.03
NTCA	…	…	…	0.51*	0.16*
Nitrate	…	…	…	…	0.31*

* 2-sided *P’*s < 0.001.

† Two hundred and sixty one controls with missing value of nitrite were excluded from the analysis.

Table 3 shows geometric means of urinary NOCs, nitrite, and nitrate among control subjects. Regular alcohol drinkers doubled their levels of urinary NMTCA relative to nondrinkers. The association was dose-dependent; the geometric means of NMTCA were 1.70 (μg/g creatinine) for non-drinkers, 3.75 for <2 drinks/day and 4.21 for ≥2 drinks/day (*P* for trend <0.0001). Smokers or those positive for antibodies to *H*. *pylori* were associated with statistically borderline significant elevation of urinary nitrite. There was no significant relation for other urinary NOCs or their precursors with alcohol intake, cigarette smoking or history of *H*. *pylori* infection (Table 3). We also examined the association between intake frequencies of fresh dark green vegetables or preserved foods and urinary levels of NOCs or their precursors among control subjects. High intake frequency of total preserved meat, fish and eggs combined was associated with high levels of urinary NMTCA (*P* for trend = 0.032). There was no statistically significant association between dietary intakes of fresh/preserved vegetables, or preserved meat/fish/egg and urinary levels of nitrite or other NOCs (Table 3).

**Table 3:** Geometric mean levels of urinary *N*-nitroso compounds by status of drinking, smoking and *H*. *pylori* serologic status among control subjects, The Shanghai Cohort Study.

		No.	NMTCA (μg/g Cr)	NPRO (μg/g Cr)	NSAR (μg/g Cr)	NTCA (μg/g Cr)	Nitrate (mg/g Cr)	Nitrite* (mg/g Cr)
Alcohol drinking	No	325	1.70 (1.34–2.12)	5.38 (4.68–6.18)	0.38 (0.28–0.48)	7.74 (6.68–8.96)	190.4 (168.5–215.1)	8.50 (7.20–10.04)
	Yes	244	3.92 (3.18–4.82)	4.88 (4.14–5.72)	0.36 (0.26–0.48)	6.86 (5.78–8.14)	191.7 (166.5–220.8)	10.08 (8.30–12.20)
	2-sided *P*		<0.0001	0.356	0.817	0.300	0.941	0.194
Cigarette smoking	Never	238	2.22 (1.72–2.82)	4.98 (4.22–5.86)	0.30 (0.20–0.40)	7.42 (6.22–8.80)	192.8 (167.2–222.4)	7.94 (6.54–9.60)
	Ever	331	2.72 (2.22–3.30)	5.30 (4.60–6.06)	0.42 (0.32–0.52)	7.32 (6.30–8.46)	189.7 (168.0–214.1)	10.2 (8.62–12.04)
	2-sided *P*		0.200	0.582	0.099	0.901	0.862	0.053
H. *Pylori* Status†	Negative	94	2.66 (1.80–3.80)	4.92 (3.82–6.28)	0.34 (0.16–0.52)	6.82 (5.18–8.92)	166.5 (133.7–207.2)	7.00 (5.20–9.30)
	Positive	447	2.50 (2.08–2.96)	5.08 (4.54–5.70)	0.38 (0.30–0.48)	7.48 (6.60–8.46)	191.4 (173.1–211.6)	9.64 (8.40–11.04)
	2-sided *P*		0.745	0.820	0.589	0.547	0.257	0.048
Intake of dark green vegetables	<1/week	45	3.06 (1.74–5.02)	4.92 (3.32–7.10)	0.60 (0.32–0.92)	6.90 (4.56–10.22)	161.0 (115.9–223.6)	7.12 (4.26–11.54)
	<1/day	273	2.42 (1.92–3.02)	5.10 (4.38–5.92)	0.34 (0.26–0.46)	7.80 (6.64–9.16)	188.9 (165.3–215.9)	10.36 (8.68–12.34)
	1/day	192	2.44 (1.84–3.16)	5.38 (4.50–6.44)	0.40 (0.28–0.54)	6.94 (5.70–8.42)	195.7 (166.9–229.4)	7.70 (6.14–9.62)
	2+/day	59	2.66 (1.6–4.16)	4.90 (3.48–6.76)	0.18 (0.00–0.38)	7.04 (4.92–9.92)	211.2 (158.6–281.3)	9.92 (6.68–14.52)
2-sided *P* for trend			0.845	0.854	0.111	0.607	0.248	0.664
Intake of salted/ pickled vegetables	None	121	2.44 (1.70–3.36)	5.28 (4.18–6.60)	0.46 (0.30–0.64)	7.52 (5.88–9.54)	232.9 (191.0–284.0)	8.88 (6.60–11.86)
	<1/month	76	3.00 (1.96–4.42)	5.86 (4.38–7.72)	0.42 (0.24–0.64)	7.08 (5.18–9.56)	200.0 (155.7–256.9)	8.14 (5.78–11.31)
	<1/week	237	2.40 (1.86–3.04)	5.32 (4.52–6.24)	0.34 (0.24–0.46)	7.62 (6.40–9.04)	197.7 (171.5–227.8)	8.76 (7.20–10.64)
	1/week	92	2.12 (1.36–3.10)	4.30 (3.26–5.60)	0.30 (0.14–0.48)	5.92 (4.42–7.84)	141.8 (112.9–178.2)	9.94 (7.26–13.48)
	2+/week	43	3.38 (1.94–5.56)	4.80 (3.22–7.00)	0.30 (0.06–0.56)	9.66 (6.46–14.26)	157.1 (112.5–219.2)	12.82 (8.12–19.98)
2-sided *P* for trend			0.936	0.297	0.125	0.961	0.002	0.211
Intake of preserved meats, salted fish and preserved eggs	<1/month	44	1.80 (0.90–3.18)	4.48 (3.00–6.54)	0.32 (0.10–0.60)	6.08 (3.96–9.08)	203.3 (145.9–283.2)	10.80 (6.90–16.62)
	<1/week	241	2.28 (1.76–2.88)	5.44 (4.62–6.38)	0.36 (0.24–0.46)	7.74 (6.52–9.18)	185.2 (160.7–213.4)	8.62 (7.06–10.46)
	1–2/week	220	2.62 (2.04–3.32)	5.18 (4.36–6.12)	0.46 (0.34–0.58)	7.28 (6.06–8.70)	205.3 (177.0–238.1)	9.28 (7.54–11.38)
	3+/week	64	3.70 (2.38–5.52)	4.60 (3.30–6.26)	0.18 (0–0.38)	7.14 (5.06–9.92)	160.1 (121.6–210.9)	9.54 (6.58–13.64)
2-sided *P* for trend			0.032	0.741	0.773	0.932	0.659	0.960

* Two hundred and sixty one controls with missing value of nitrite were excluded from the analysis.

† Determined by serologic status of *H*. *pylori* antibodies. Twenty eight controls with missing *H*. *pylori* status were excluded from the analysis.

High intake of salted/pickled vegetables was inversely associated with urinary nitrate level in a dose-dependent manner (*P* for trend = 0.002) whereas high intake of fresh dark green vegetables was associated with a statistically non-significant increase in nitrate level overall (*P* for trend = 0.248), although there was no correlation between intake frequencies of fresh and preserved vegetables (r = 0.02, *P* = 0.58). We examined the modifying effect of salted/pickled vegetables on the association between intake of fresh dark green vegetables and urinary levels of nitrate. Among controls who did not consume salted/pickled vegetables, the geometric means (95% CIs) of urinary nitrate for individuals consuming <1, 1, and 2 or more times/day of fresh dark green vegetables were 206.0 (151.5–280.0), 201.3 (144.4–280.5), and 523.0 (274–995.8) mg/g creatinine, respectively (*P* for trend = 0.069). Among controls who consumed one or more times of salted or pickled vegetables per week, the corresponding figures were 141.9 (110–181.7), 154.8 (117.9–203.1), and 120.0 (70.9–202.6) mg/g creatinine, respectively (*P* for trend = 0.84). There was a statistically significant interaction between intakes of fresh dark green vegetables and salted/pickled vegetables on urinary level of nitrate (P for interaction = 0.037).

In the study population, the median levels of urinary nitrate (170–190 mg/g creatinine) were more than 20 times those of nitrite (7–8 mg/g creatinine) in both cases and controls. Among four NOCs measured, NTCA was at the highest level, followed by NPRO and NMTCA whereas NSAR was at the lowest; more than 60% of study subjects were undetectable for NSAR in urine. Overall there was no significant difference in urinary concentrations of nitrate, nitrite or any NOCs measured between gastric cancer patients and control subjects (Table 4). Compared with low levels (i.e., undetectable or lowest tertile), highest levels of individual NOCs or their precursors were not associated with increased risk of gastric cancer in all subjects after adjustment for alcohol consumption, cigarette smoking, *H*. *pylori* serologic status, antioxidant measurements which included urinary EGC and serum β-carotene and vitamin C (Table 5).

**Table 4:** Median (range) levels of urinary *N*-nitroso compounds in gastric cancer cases and control subjects, The Shanghai Cohort Study.

*N*-nitroso compounds	Cases (n = 191) (5^th^-95^th^ percentile)	Controls (n = 569) (5^th^-95^th^ percentile)	2-sided *P* *
NMTCA (μg/g Cr)	2.22 (0–39.87)	2.11 (0–36.52)	0.689
NPRO (μg/g Cr)	4.52 (0.59–20.29)	4.30 (0–42.94)	0.892
NSAR (μg/g Cr)	0 (0–2.67)	0 (0–4.65)	0.872
NTCA (μg/g Cr)	5.63 (0–48.88)	7.26 (0–72.78)	0.203
Nitrate (mg/g Cr)	169.1 (49.4–684.7)	190.0 (40.9–1189.3)	0.558
Nitrite (mg/g Cr) †	7.41(2.15–71.49)	8.08 (1.95–72.85)	0.342

* Derived from Wilcoxon test.

† Eighty seven cases and two hundred and sixty one controls with missing value of nitrite were excluded from the analysis.

**Table 5:** Levels of urinary *N*-nitroso compounds in relation to risk of gastric cancer, The Shanghai Cohort Study.

	Low	Intermediate	High	2-sided *P* for trend
NMTCA (μg/g Cr)	0	≤ 6.79	> 6.80	
Cases/Controls	85/249	57/161	49/159	
OR (95% CI)*	1.00	1.11 (0.72–1.71)	0.82 (0.54–1.26)	0.415
NPRO (μg/g Cr)	≤ 2.60	2.61–7.51	> 7.51	
Cases/Controls	58/189	75/195	58/185	
OR (95% CI)*	1.00	1.29 (0.85–1.97)	0.99 (0.64–1.52)	0.936
NSAR (μg/g Cr)	0	≤ 0.77	> 0.78	
Cases/Controls	126/371	26/97	39/101	
OR (95% CI)*	1.00	0.75 (0.43–1.29)	1.19 (0.74–1.93)	0.644
NTCA (μg/g Cr)	≤ 3.63	3.64–12.16	> 12.16	
Cases/Controls	66/189	70/195	55/185	
OR (95% CI)*	1.00	1.03 (0.68–1.56)	0.82 (0.54–1.25)	0.346
Nitrate (mg/g Cr)	≤ 114.9	115.0–285.5	> 285.5	
Cases/Controls	54/187	83/197	54/185	
OR (95% CI)*	1.00	1.37 (0.91–2.09)	1.05 (0.67–1.63)	0.859
Nitrite (mg/g Cr) †	≤ 5.30	5.31–11.87	> 11.87	
Cases/Controls	40/103	23/104	41/101	
OR (95% CI)*	1.00	0.62 (0.32–1.22)	1.16 (0.59–2.30)	0.642

* Odds ratios were calculated using conditional logistic models adjusted for education levels (no or primary school, middle school, high school and above), alcohol consumption (non-drinker, <4 drinks/day, 4+ drinks/day), smoking status (never *vs*. ever smokers), levels of serum vitamin C (below *vs*. above median), serum β-carotene (below *vs*. above median), and urinary epigallocatechin (below *vs*. above median) and *H*. *pylori* status (negative *vs*. positive). To maximize sample size, an indicator variable was created separately for missing values of serum vitamin C (5 cases), serum β-carotene (2 cases and 3 controls), urinary epigallocatechin (5 cases and 20 controls), and *H*. *pylori* (9 cases and 28 controls).

† Eighty seven cases and two hundred and sixty one controls with missing value of nitrite were excluded from the analysis.

Given that infection with *H*. *pylori* is an underlying cause of gastric cancer, we also examined the associations between urinary levels of nitrate, nitrite and individual NOCs and risk of gastric cancer separately in individuals positive or negative for *H*. *pylori* antibodies (Table 6). Among individuals negative to *H*. *pylori* antibodies, elevated urinary nitrate was associated with a statistically significant increased risk of gastric cancer; the multivariate-adjusted ORs (95% CIs) for the second and third tertiles of nitrate were 3.27 (0.76–14.03) and 4.82 (1.05–22.17), respectively, compared with the lowest tertile (*P* for trend = 0.042). There was no evidence for effect modification of *H*. *pylori* serologic status on the association between nitrite or any NOCs and risk of gastric cancer (Table 6). We also examined and found no modifying effect of cigarette smoking, alcohol consumption, levels of serum β-carotene and vitamin C, and urinary EGC on the association between NOCs or their precursors and gastric cancer risk (data not shown).

**Table 6:** Urinary level of *N*-nitroso compounds in relation to the risk of gastric cancer stratified by *H*. *pylori* serologic status.

*N-*nitroso-compound	H. pylori negative*		H. pylori positive*	
	Cases/Controls	OR† (95% CI)	Cases/Controls	OR† (95% CI)
NMTCA				
Low	7/37	1.00	72/196	1.00
Intermediate	9/31	1.37 (0.42–4.47)	47/127	1.09 (0.69–1.72)
High	4/26	0.60 (0.14–2.53)	43/124	0.92 (0.58–1.45)
2-sided *P* for trend		0.556		0.761
NPRO				
Low	6/29	1.00	48/152	1.00
Intermediate	10/36	1.32 (0.38–4.54)	60/150	1.28 (0.81–2.03)
High	4/29	0.56 (0.13–2.47)	54/145	1.21 (0.76–1.92)
2-sided *P* for trend		0.454		0.437
NSAR				
Low	17/58	1.00	105/292	1.00
Intermediate	1/19	0.16 (0.02–1.37)	23/75	0.89 (0.52–1.53)
High	2/17	0.28 (0.05–1.56)	34/80	1.21 (0.75–1.95)
2-sided *P* for trend		0.062		0.541
NTCA				
Low	6/33	1.00	57/144	1.00
Intermediate	10/38	1.17 (0.36–3.81)	55/153	0.89 (0.57–1.40)
High	4/23	0.74 (0.17–3.22)	50/150	0.84 (0.53–1.33)
2-sided *P* for trend		0.738		0.464
Nitrate				
Low	3/35	1.00	47/144	1.00
Intermediate	9/34	3.27 (0.76–14.03)	72/156	1.38 (0.88–2.16)
High	8/25	4.82 (1.05–22.17)	43/147	0.93 (0.57–1.52)
2-sided *P* for trend		0.042		0.792
Nitrite‡				
Low	3/24	1.00	36/76	1.00
Intermediate	3/23	1.59 (0.22–11.40)	20/80	0.56 (0.29–1.11)
High	3/12	1.65 (0.15–17.59)	37/87	1.03 (0.55–1.93)
2-sided *P* for trend		0.633		0.939

* Thirty seven subjects (9 cases and 28 controls) were excluded due to missing *H*. *pylori* status.

† Odds ratios were calculated using conditional logistic models adjusted for education levels (no or primary school, middle school, high school and above), alcohol consumption (non-drinker, <4 drinks/day, 4+ drinks/day), smoking status (never *vs*. ever smokers), levels of serum vitamin C (below *vs*. above median), serum β-carotene (below *vs*. above median), and urinary epigallocatechin (below *vs*. above median) and *H*. *pylori* status (negative *vs*. positive). To maximize sample size, an indicator variable was created separately for missing values of serum vitamin C (4 cases), serum β-carotene (1 cases and 2 controls), and urinary epigallocatechin (5 cases and 18 controls).

‡ Eighty cases and two hundred thirty nine controls with missing value of nitrite were excluded from analysis.

The association between urinary levels of nitrate, nitrite or NOCs and risk of cardia or non-cardia cancer was examined. Overall no statistically significant association was found for urinary NOCs or their precursors with risk of either cardia or non-cardia cancer (data not shown).

## Discussion

To the best of our knowledge, the present study was the first utilizing a biomarker approach to investigate the association between NOCs and their precursors and the risk of developing gastric cancer in a prospective cohort study. We observed a statistically significant positive association between urinary nitrate and gastric cancer risk among individuals negative for IgG antibodies to *H*. *pylori* (i.e., no history of infection with *H*. *pylori*). In addition, elevated levels of urinary nitrite were associated with seropositivity to *H*. *pylori*, suggesting that infection with *H*. *pylori* may enhance the reduction of nitrate to nitrite *in vivo*. Furthermore, the present study demonstrated a novel dose-dependent relationship between alcohol consumption and urinary levels of NMTCA, suggesting that alcohol intake could play a role in the formation of NOCs *in vivo*.

Infection with *H*. *pylori* is a strong risk factor for gastric cancer [33]. The presence of *H*. *pylori* in the stomach causes inflammatory damage to the mucosa of the stomach, which may enhance the endogenous formation of NOCs in the stomach [18, 34, 35]. An elevated level of urinary nitrite in individuals seropositive for antibodies to *H*. *pylori* supports this hypothesis that *H*. *pylori* may contribute to nitrosation process in the stomach. The interactive role of *H*. *pylori* and *N*-nitroso compounds in the development of gastric cancer requires further investigation.

Among individuals seronegative for IgG antibodies to *H*. *pylori*, urinary levels of nitrate, a precursor of *N*-nitroso compounds, were positively associated with risk of gastric cancer. Chronic infection with *H*. *pylori* is an established risk factor for gastric cancer. It is not surprising that the association between nitrate and gastric cancer risk was more apparent among individuals without *H*. *pylori* infection, a relatively low risk population. The findings of the present study suggest that the effect of nitrate through the nitrosation pathway could be masked by *H*. *pylori* infection, especially in the present study population where *H*. *pylori* infection is highly prevalent. Given the small sample size and multiple comparison issues, the interpretation of the present study findings with a positive association between urinary nitrate and gastric cancer risk among individuals with negative *H*. *pylori* should be made with caution because of possible chance finding. The future studies with a larger sample size are warranted to confirm these results.

This study shows that higher levels of NMTCA in the urine of alcohol drinkers compared to non-drinkers. Studies have shown that co-administration of acetaldehyde (a metabolite of ethanol), L-cysteine and nitrite significantly increased urinary excretion of NMTCA in man [24]. The present study demonstrated for the first time in free-living individuals that alcohol consumption resulted in a significantly increased level of urinary NMTCA. High intake of preserved meats (including salted pork, cured meat, sausage and ham), salted fish and preserved eggs also was associated with elevated levels of NMTCA (*P* for trend = 0.03). Our findings were consistent with the result from a recent study showing that NMTCA were often detected in processed meat products [36, 37]. These findings may shed some light on the mechanism of consumption of alcohol and preserved meat/fish products in the formation of certain NOCs *in vivo* and may explain their association with risk of gastric cancer.

The present study demonstrated a strong, statistically significant inverse association between intake of salted/pickled vegetables and urinary levels of nitrate (*P* = 0.002). In addition, a statistically significant modifying effect of salted/pickled vegetables on the relationship between dark green vegetables and urinary nitrate level was found. Intake of salted/pickled vegetables resulted in a statistically non-significant 20–30% reduction in urinary nitrate level among individuals with less than two times/day of dark green vegetables whereas it resulted in a statistically significant 77% decrease in urinary nitrate level among those consuming two or more times of fresh dark green vegetables (P for interaction = 0.037). The fermentation or pickling process can significantly reduce the amount of nitrate in salted/pickled vegetables by up to 90% [38, 39]. This effect could be due to the reduction of nitrate to nitrite that could be catalyzed by bacteria containing nitrate reductase[40]. The findings of the present study suggested that nitrate reductase in bacteria present in salted/pickled vegetables may reduce nitrate derived from fresh dark green vegetables in oral cavity and/or stomach.

In the present study, we found that several NOCs levels in urine were correlated with each other. NTCA and NPRO had the highest correlation and both were significantly correlated with nitrate. Due to the complexities of the assays for multiple NOCs in a single batch run, limited data on the relationships among NOCs on an individual basis have been reported. According to a recent survey on the food sources of NOCs NTCA and NPRO are two of the most frequently assayed NOCs and share similar food sources such as processed meat products [37]. The *in vivo* administration of nitrate and their respective secondary amine precursor has shown high efficiency in the production of endogenous NTCA and NPRO [22, 23], resulting in their correlation with nitrate.

Nitrate and nitrite are classified as probably carcinogenic to humans under conditions likely to cause endogenous nitrosation [8]. A positive association between dietary intake of nitrites and nitrosamines and gastric cancer risk has been observed in several case-control studies [41–44]. The consumption of processed meat, preserved fish and preserved vegetables was associated with increased risk of gastric cancer with an OR being from 1.10 to 5.51 [20]. However, several cohort studies showed inconsistent results. For instance, a large multicenter European cohort study reported that individuals with the highest propensity for endogenous NOCs formation had an elevated risk for gastric cancer [45]. In contrast, a large Finnish cohort study found no association between estimated intake of nitrites, nitrates and *N*-nitrosodimethylamine and risk of gastric cancer [46]. In the present study, we found a statistically significant, positive association between prediagnostic urinary nitrate level and risk of gastric cancer among *H*. *pylori*-negative individuals. The lack of an overall positive association between urinary levels of selected NOCs and their precursors and risk of gastric cancer could be due to the high prevalence of *H*. *pylori* infection in the study population; 82.6% of control subjects had a history of infection with *H*. *pylori* (i.e., positive for IgG antibodies to *H*. *pylori*). The strong effect of *H*. *pylori* on gastric carcinogenesis could mask the role of NOCs or their precursors in the risk of gastric cancer development in this study population.

Our study had several strengths. A prospective study design and the availability of prediagnostic urine samples minimized the possible influence of disease symptoms on dietary intake and other lifestyle factors. The prospective design of the study also ruled out the possibility of recall bias on exposure. Considering the limitation of food frequency questionnaire in the estimation of exposure levels to both endogenous and exogenous NOCs [19], urinary levels of NOCs as exposure biomarkers may overcome such limitations and capture both sources of NOCs. The simultaneous adjustment for cigarette smoking, alcohol drinking, *H*. *pylori* infection, and laboratory measurements of antioxidants (i.e., serum β-carotene and vitamin C and urinary EGC) could reduce their potential confounding effect on the NOCs-gastric cancer risk associations. The almost complete follow-up for incident cancer and death minimized the potential bias on results due to the loss to follow-up.

The present study has several potential limitations. One cannot presume that NOCs levels in a randomly timed, single void urine sample represented the long-term exposure to both exogenous and endogenous NOCs. It would be ideal, but rarely feasible, to assess NOC exposure at multiple time points prior to disease occurrence, especially using a biomarker approach as this study, due to the high cost and logistical complexity involved in the collection of biospecimens at multiple points of time for each subject from a prospective study of large number of participants such as this one. Therefore, the non-differential misclassification due to the use of a single spot urine sample may be the underlying reason for the null findings of urinary NOCs and gastric cancer risk. Another major limitation of the present study was the relatively small sample size, especially those negative for *H*. *pylori* antibodies in subgroup analysis with unconditional logistic regression after the matched case-control sets were broken, which prohibited us from conducting further stratified statistical analyses.

## Conclusions

In summary, the present study did not show an overall statistically significant association between urinary levels of selected NOCs or their precursors and risk of gastric cancer in a high-risk population with high prevalence of *H*. *pylori*. A statistically significant positive association between prediagnosic urinary levels of nitrate and risk of gastric cancer was observed among individuals negative for antibodies to *H*. *pylori*. This positive nitrate-gastric cancer risk association was independent of cigarette smoking, alcohol consumption and antioxidant status. The present study also found a strong, dose dependent relationship for the consumption of alcohol or preserved meats and fishes with urinary levels of NMTCA, which sheds some light on our understanding the mechanism of alcohol and preserved protein-rich food items on gastric carcinogenesis. The role of *N*-nitroso-compounds in the development of gastric cancer in humans is warranted for further investigation.
